# Complexity in disease management: A linked data analysis of multimorbidity in Aboriginal and non-Aboriginal patients hospitalised with atherothrombotic disease in Western Australia

**DOI:** 10.1371/journal.pone.0201496

**Published:** 2018-08-14

**Authors:** Mohammad Akhtar Hussain, Judith M. Katzenellenbogen, Frank M. Sanfilippo, Kevin Murray, Sandra C. Thompson

**Affiliations:** 1 Western Australian Centre for Rural Health, The University of Western Australia, Geraldton, Western Australia, Australia; 2 School of Population and Global Health, The University of Western Australia, Perth, Western Australia, Australia; University of Oxford, UNITED KINGDOM

## Abstract

**Background:**

Hospitalisation for atherothrombotic disease (ATD) is expected to rise in coming decades. However, increasingly, associated comorbidities impose challenges in managing patients and deciding appropriate secondary prevention. We investigated the prevalence and pattern of multimorbidity (presence of two or more chronic conditions) in Aboriginal and non-Aboriginal Western Australian residents with ATDs.

**Methods and findings:**

We used population-based de-identified linked administrative health data from 1 January 2000 to 30 June 2014 to identify a cohort of patients aged 25–59 years admitted to Western Australian hospitals with a discharge diagnosis of ATD. The prevalence of common chronic diseases in these patients was estimated and the patterns of comorbidities and multimorbidities empirically explored using two different approaches: identification of the most commonly occurring pairs and triplets of comorbid diseases, and through latent class analysis (LCA). Half of the cohort had multimorbidity, although this was much higher in Aboriginal people (Aboriginal: 79.2% vs. non-Aboriginal: 39.3%). Only a quarter were without any documented comorbidities. Hypertension, diabetes, alcohol abuse disorders and acid peptic diseases were the leading comorbidities in the major comorbid combinations across both Aboriginal and non-Aboriginal cohorts. The LCA identified four and six distinct clinically meaningful classes of multimorbidity for Aboriginal and non-Aboriginal patients, respectively. Out of the six groups in non-Aboriginal patients, four were similar to the groups identified in Aboriginal patients. The largest proportion of patients (33% in Aboriginal and 66% in non-Aboriginal) was assigned to the “minimally diseased” (or relatively healthy) group, with most patients having less than two conditions. Other groups showed variability in degree and pattern of multimorbidity.

**Conclusion:**

Multimorbidity is common in ATD patients and the comorbidities tend to interact and cluster together. Physicians need to consider these in their clinical practice. Different treatment and secondary prevention strategies are likely to be useful for management in these cluster groups.

## Introduction

Multimorbidity, often defined as the presence of two or more chronic conditions in an individual, is not just common in the elderly, it is also a problem in younger adults [1, 2]. Multimorbidity is associated with decreased quality of life, functional impairment, increased health care utilization, polypharmacy, increased workload for self-management and increased mortality [3–8]. Consequently, it has important implications in health service planning with its impact on health care resources likely to differ across health systems, regions, disease combinations and person-specific factors, including social disadvantage and age [9].

Cardiovascular disease (CVD) accounts for the majority of the mortality and morbidity burden in Australia, with almost 20% of all Pharmaceutical Benefits Scheme (PBS) medicines dispensed in 2014–15 being for CVD [10]. The majority of these CVDs are attributable to atherothrombotic diseases (ATDs), including cerebrovascular disease (CeVD, including stroke), coronary heart disease (CHD), and peripheral arterial disease (PAD), which share a common metabolic etiology and are associated with increased risk of atherothrombotic events. The burden of ATDs is significantly higher in older age groups. However, it is an emerging challenge for the younger population as well, and is predicted to rise further in coming decades [11–13]. Additionally, ATDs carry a high burden of comorbidities which have negative consequences on health services as well as on patient health outcomes by increasing the risk of recurrent CVD events and associated mortality [14, 15].

The Western Australian (WA) population is broadly similar to the Australian population in terms of socioeconomic and demographic characteristics [16]. In its population of 2.5 million, almost 3.0% are Aboriginal and Torres State Islander people (hereafter respectfully referred to as Aboriginal) [16]. Aboriginal Australians experience a significantly higher burden of CVDs across all age groups with an earlier age of onset compared to other Australians [11, 17–20]. The relative disparity in incidence of hospitalised stroke, myocardial infarction, atrial fibrillation and heart failure is also extremely high among younger adults, reducing with age although absolute differences in rates remain very high at older ages as well [19, 21–24]. Aboriginal adults were almost twice as likely to be hospitalised with a principal diagnosis of CVD, and more than half of Aboriginal hospitalisations for CVD occurred in people aged under 55 years compared with only 17% in non-Aboriginal people [25]. Moreover, the burden of comorbidities associated with CVDs is also substantially higher in Aboriginal people than other Australians, predominantly due to the higher prevalence of other chronic diseases [20, 25]. The presence of multiple chronic conditions increases the complexity of managing these patients with CVDs where the traditional single disease care strategy may not work. Additionally, several studies have shown that multimorbidity is not only a problem of old age [1, 2, 26] and that multimorbidity among young adults remains under-researched.

Understanding specific conditions that co-occur with ATDs is important for its management and for designing secondary prevention interventions, particularly at younger ages where the potential for prevention is the highest. Hence, research exploring specific disease combinations and common clusters of diseases rather than simple disease counts is likely to provide useful insights into the complex care needs of individuals with multi-morbidity [27, 28]. The majority of contemporary studies focus on differences in the prevalence of comorbidities and their impact on outcome rather than patterns of co-morbidity [29–31]. Our aim was to identify the prevalence and patterns of multimorbidity in WA residents under 60 years of age with ATDs admitted to public and private hospitals and examine differences by Aboriginality. This was based on knowledge of the existing disparities in the disease incidence, prevalence and hospitalisation rate in the Aboriginal and non-Aboriginal population, with the expectation that the pattern of multimorbidity is likely to vary by Aboriginality.

## Methods

### Study design and data sources

This descriptive study used a hospital ATD cohort identified using linked administrative health data. Population-based de-identified administrative health data from 1 January 2000 to 30 June 2014 were extracted from the Hospital Morbidity Data Collection (HMDC) and Mortality database, two of the core datasets of the WA Data Linkage System (WADLS) [32].

### Study cohort and identification of ATD

We defined ATD as ischaemic cardiovascular disease encompassing coronary, cerebral or peripheral territories (not mutually exclusive). Given the younger age profile of the Aboriginal population, the 10-year life expectancy gap between Aboriginal and non-Aboriginal Australian populations and the early onset of ATD among Aboriginal people [33], a prevalent cohort (age range 25–59 years) was identified consisting of all residents ever hospitalised with an ATD discharge diagnosis in Western Australia (WA) during the period 1 January 2000 to 30 June 2014 and still alive at 30 June 2014.

International Classification of Diseases and Related Health Problems 10^th^ revision Australian Modification (ICD-10-AM) codes pertaining to ATDs were used to identify emergency and elective hospital admissions with discharge diagnosis in any of the 22 diagnosis fields during the 14.5-year time period. The prevailing ATDs were identified using previously validated codes (CHD: I20–I25; CeVD: I61, I63, I64, I65, I66, I69, G45; PAD: I70, I73.1, I73.9, I77.1) [29], allowing capture of virtually all prior ATD hospitalisations [30].

### Identification of comorbidities

Similar to ATDs, co-occurring chronic morbidities were determined from each patient’s hospital records dating back to 1 January 2000 from 30 June 2014. Comorbidities were identified if they were coded in one of 22 diagnosis fields for any hospitalisation during the study period. For the purpose of this study, we focused on the 18 most common chronic comorbid conditions with prevalence (per 100 ATD patients) ≥3% [28] (**S1 Table**). The selection was also informed by the results of previous studies, the Australian Burden of Disease Study and expert opinion [18, 34, 35]. To identify cancer diagnoses, we considered 5-year prevalence which represents patients alive on 30 June 2014 and diagnosed with a cancer within the previous five years, after 30 June 2009 [36, 37]. We considered this five year cancer survival period as this is the time with the highest demands for health care needs, including diagnosis, initial treatment, and most intensive follow-up [36]. For diagnosis of psychiatric comorbidities, we considered the variability in recording of psychiatric morbidities in patients with multiple treatment episodes and followed a diagnostic hierarchy to identify the most relevant psychiatric comorbidity [38, 39]. Comorbidities were identified individually and later grouped for exploring the pattern of coexistence in individual ATD patients.

### Cohort characteristics

Demographic characteristics of the cohort including age, gender, type of hospital admission (emergency/booked), and hospital type were identified from the hospital records. To account for under-identification of Aboriginal status in administrative health data [40, 41], Aboriginality was ascertained by the WADLS using a method using multiple administrative data sources described by Christensen et al [42]. Socio-economic status (SES) of individual patients was based on the quintiles of Socio-Economic Indexes for Areas (SEIFA), an area-level relative socio-economic disadvantage score derived from the census [43]. Residential postcode was used to categorise patients into the five levels of Accessibility/Remoteness Index of Australia (ARIA) [44].

### Data analysis

#### Profile of patients and ATD prevalence

All analyses were stratified based on the Aboriginality status of the admitted patient with demographic and comorbidity characteristics summarised separately for Aboriginal and non-Aboriginal patients. We calculated the age- and sex-standardised point prevalence of ever hospitalised ATDs in the general population based on the age and sex structure (2014 mid-year) of the WA population (Aboriginal and non-Aboriginal), sourced from the Epidemiology Branch, WA Department of Health.

For each comorbid chronic disease, the prevalence and standard error (SE) and the mean number and SE of co-occurring conditions were estimated. All prevalence estimates were age-standardised using the WA 2014 mid-year population and reported along with their 95% confidence intervals (CI).

#### Multimorbidity

Multimorbidity was defined as the presence of two or more chronic conditions (in addition to ATD). Univariate analyses were carried out to determine the distribution of multimorbidity across selected socio-demographic characteristics: age, gender, SEIFA quintiles and ARIA. We used the Stata command “*binreg*” to fit a generalized linear model (GLM) for binary outcomes with log link function to explore patient characteristics predicting multimorbidity, with results being presented as risk ratios (RR) [45].

To determine the pattern of multimorbidity among the ATD cohort, two different approaches were used: the ratio of observed (O) over expected (E) prevalence (O/E ratio), and latent class analysis (LCA). These methods explored whether diseases are independent of each other, and if not, whether there are any common patterns of grouping across different approaches.

Firstly, for estimating the O/E ratios, we explored all possible combinations (duplets and triplets) of comorbid conditions. Later, we calculated the observed prevalence (a percentage of the ATD cohort) of each combination of chronic diseases. The prevalence of the ten most common disease combinations (duplets and triplets) was ascertained and compared with the expected prevalence of those combinations [46–48]. The expected prevalence of the combinations of chronic diseases was based on the assumption that they were statistically independent of one another, and was calculated by multiplying the prevalence of one condition by the second [28]. The O/E ratios were calculated to examine whether conditions were more or less likely than statistical chance to occur together. The statistical independence of two diseases included in a disease pair was tested using a chi-squared test. Additionally, the statistical independence between each pair of co-occurring diseases was also tested through logistic regression models after adjusting for age, gender, ARIA, SEIFA index quintiles and all of the other diseases [28]. The mean and standard error of the number of co-occurring chronic diseases (apart from the ATDs) were estimated.

We performed LCA to identify a set of discrete, mutually exclusive clusters or latent classes of individuals based on their responses to a set of observed categorical variables [49]. The 18 individual comorbidities were used in LCA to empirically identify latent multimorbidity clusters. We explored a sequence of LCA models without any *a priori* assumptions about the number of latent classes. We started with a two-class model and increased up to seven classes in a stepwise fashion [49]. The model selection was based on a balance of parsimony, substantive consideration, and several fit indices [49]. The Akaike Information Criterion (AIC), the Bayesian Information Criterion (BIC) and Consistent AIC (cAIC) were used to measure the relative fit of the models [49, 50]. In addition, model interpretability was considered. For example, distinguishability of each class from the others on the basis of the item-response probabilities, triviality in size (none near zero), and the possibility of assigning a meaningful label to each class [49]. After selecting a latent class model, we assigned each participant to his or her “best fit” class (based on the highest computed probability of membership) [49]. Each latent class was labelled according to those chronic conditions where prevalence exceeded the prevalence in the full cohort [51]. In the next step, multivariate, multinomial logistic regression was used to identify the predictor of the group member probability, with results presented in the form of relative risk ratios (RRR). For parameter estimation and model selection in LCA, we used an iterative maximum likelihood estimation method with ‘random’ starting values. The validity of the final fit model was also tested by repeating the estimation process with a different set of ‘random’ starting values, and the solutions were considered to be identical if the log likelihood and parameter estimates were replicated [49].

All analyses were performed using STATA Version-14.0 (StataCorp) and SAS for Windows (Version 9.4).

### Ethics

Approval for the study was obtained from the Western Australian Department of Health Human Research Ethics Committee, the University of Western Australia Human Research Ethics Committee and the Western Australian Aboriginal Health Ethics Committee.

## Results

We identified 18,194 prevalent ATD patients, of which 11.6% (N = 2,106) were Aboriginal, 67.9% were male, 72% lived in metropolitan areas and 10% in remote and very remote areas (**S2 Table**). The mean (± standard deviation, SD) age of the study sample was 51.2 years (± 6.7) with Aboriginal patients significantly younger than non-Aboriginal. Around one in four patients were admitted to tertiary hospitals, half to other metro hospitals (of which 63% were in private hospitals) and a quarter to rural hospitals.

### Prevalence of hospitalized ATDs, comorbid chronic conditions, and burden of multimorbidity

The age-standardized prevalence of hospitalized ATDs in the Aboriginal population group was almost five times higher than in non-Aboriginals (6,306 vs. 1,305 per 100,000 population, respectively). Overall, hypertension (40.6%), diabetes (19.7%), acid peptic diseases (17.5%), alcohol abuse (16.5%) and other substance abuse (11.3%) were the comorbidities with prevalence greater than 10% (Table 1). The prevalence of comorbidities was higher in women except for hypertension and alcohol abuse disorder (**S1 Fig**).

**Table 1:** Age-standardised prevalence of chronic conditions included in the estimation of multimorbidity with associated number of co-occurring conditions.

		Aboriginal Cohort			Non-Aboriginal Cohort		
		(N = 2,106)			(N = 16,088)		
	Chronic Disease	Prevalence % (SE)	Cases without multimorbidities (%)	Mean (±SE) number of co-occurring conditions	Prevalence % (SE)	Cases without multimorbidity (%)	Mean (±SE) number of co-occurring conditions
1	Hypertension	59.8 (1.8)	7.1	3.0 (0.09)	36.7 (0.7)	30.3	1.5 (0.07)
2	Alcohol abuse disorders	47.7 (1.8)	4.1	3.2 (0.09)	9.5 (0.6)	8.4	2.4 (0.09)
3	Diabetes	47.3 (1.7)	3.3	3.5 (0.14)	14.3 (0.4)	10.6	2.0 (0.13)
4	Acid peptic disease	25.9 (1.4)	3.9	3.9 (0.12)	15.6 (0.5)	16.9	2.1 (0.11)
5	Chronic kidney disease	23.3 (1.4)	0.2	4.3 (0.13)	6.9 (0.4)	3.9	3.3 (0.15)
6	Substance Abuse disorder	19.1 (1.6)	2.5	3.5 (0.14)	9.3 (0.6)	9.2	2.6 (0.08)
7	Heart Failure	18.7 (1.4)	0.9	4.5 (0.16)	5.2 (0.3)	5.9	3.2 (0.24)
8	COPD	15.7 (1.2)	1.0	4.3 (0.20)	4.1 (0.3)	6.5	3.0 (0.18)
9	Depression	10.3 (1.1)	1.5	3.6 (0.15)	6.7 (0.4)	4.5	2.8 (0.11)
10	Asthma	9.8 (0.9)	1.7	4.5 (0.20)	2.7 (0.3)	9.5	3.1 (0.21)
11	Atrial Fibrillation	9.2 (0.9)	0.4	4.6 (0.23)	4.2 (0.2)	11.5	2.5 (0.29)
12	Valvular Heart Disease	7.6 (1.1)	1.7	4.5 (0.19)	3.8 (0.3)	10.1	2.5 (0.22)
13	Vison loss or visual disturbances	7.0 (1.1)	0.9	4.5 (0.22)	7.5 (0.6)	12.5	2.1 (0.11)
14	Rheumatoid Arthritis	6.4 (0.8)	1.8	4.9 (0.32)	4.1 (0.3)	8.9	2.7 (0.15)
15	low back pain	6.1 (0.7)	1.3	4.7 (0.19)	4.6 (0.3)	9.0	2.6 (0.19)
16	Non-skin cancer	5.8 (0.7)	4.2	3.7 (0.22)	8.2 (0.3)	16.5	1.9 (0.15)
17	Endocrine disorders (exc. diabetes)	4.6 (0.7)	5.6	4.7 (0.22)	3.3 (0.3)	7.5	2.7 (0.20)
18	Osteoarthritis	2.1 (0.2)	7.5	4.2 (0.37)	4.3 (0.2)	14.3	1.8 (0.31)

SE = standard error, COPD = chronic obstructive pulmonary disease.

Of all ATD patients, a quarter had no co-morbidities, with 28% having one, and 46% having two or more additional comorbidities (≥2 morbidities: 19.3%; ≥3 morbidities: 27.0%). Among Aboriginal patients with ADT, hypertension and osteoarthritis occurred most often in the absence of other chronic conditions (7.1% and 7.5%, respectively, **Table 1**), whereas in non-Aboriginal patients, hypertension, acid peptic diseases, non-skin cancers and osteoarthritis were most likely to occur as isolated comorbidities (30.3%, 16.9%, 16.5% and 14.3% respectively).

On average, patients with ATD had 2.0 additional comorbidities. The age-standardised mean number of comorbid conditions varied from 1.5 to 3.3 in the non-Aboriginal group and from 3.0 to 4.9 in the Aboriginal group (**Table 1**). Overall, Aboriginal patients had a significantly higher mean number of comorbidities compared to non-Aboriginal patients [3.5 (95% CI: 3.4–3.7) and 1.8 (95% CI: 1.8–1.9), respectively] and the age-standardised prevalence of multimorbidity in the Aboriginal cohort was twice that of the non-Aboriginal cohort (79.2% vs. 39.3%). Irrespective of Aboriginal status, women had significantly more comorbidities than men [Aboriginal: mean 3.6 (95% CI: 3.3–3.8) vs 3.0 (95% CI: 2.8–3.2); non-Aboriginal: mean 1.7 (95% CI: 1.6–1.8) vs 1.4 (95% CI: 1.3–1.5)].

**Fig 1:**
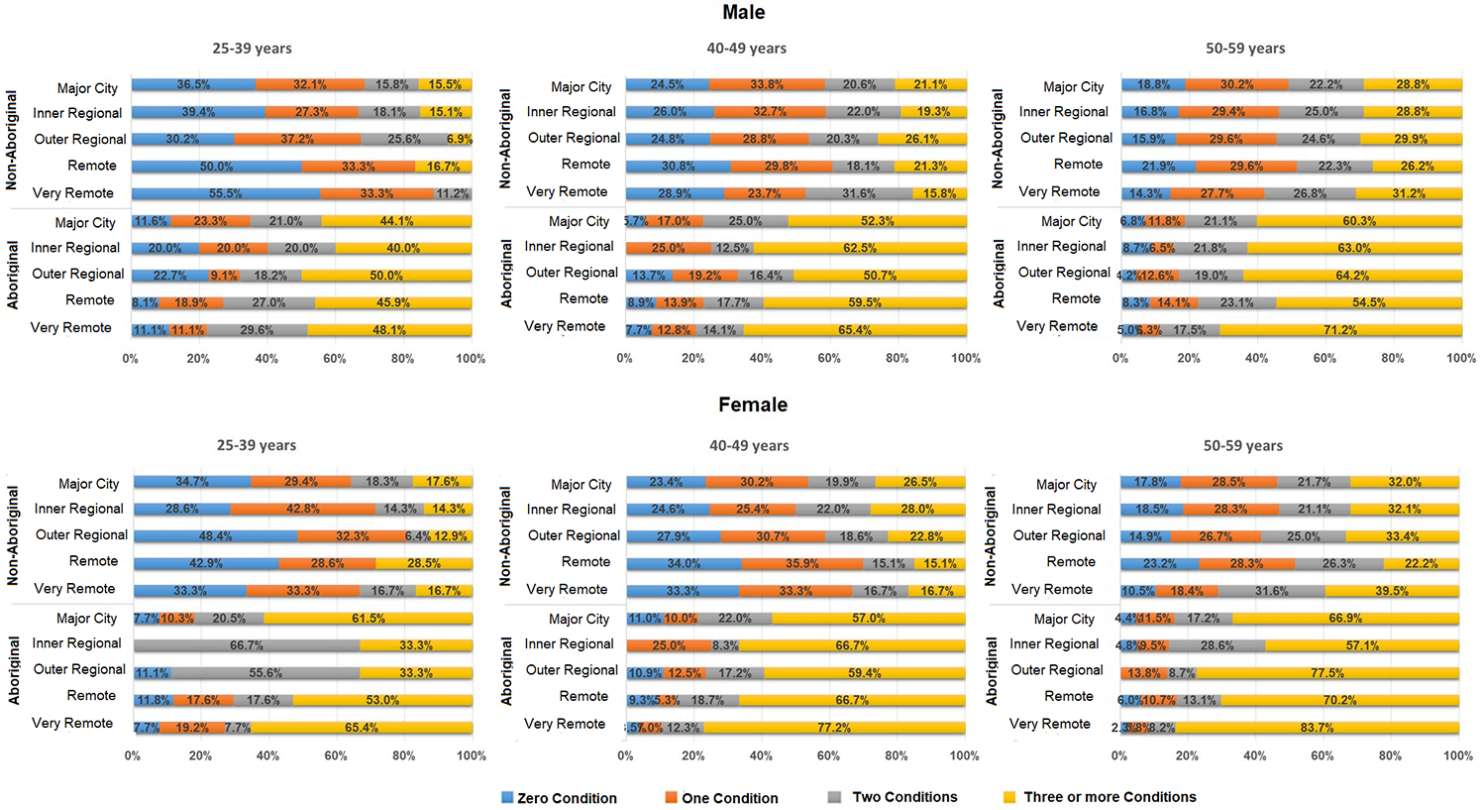
Sex stratified distribution of number of morbidities based on different age categories, Aboriginality status and their area of residence.

The prevalence of multimorbidity increased significantly from 40% (95% CI: 38.1–43.8) in the 25–39 year age group to 55% (95% CI: 53.9–55.7) in those aged 50–59 years, a trend similar across both genders (**Fig 1**). Across both Aboriginal and non-Aboriginal cohorts, women had a 10–20% greater risk of having multimorbidity and the risk increased significantly with increasing age (**Table 2**). Patients from very remote areas were 10–30% more likely to have multimorbidity than patients living in Metro areas. The level of socio-economic disadvantage (SEIFA score quintiles) did not appear to influence the risk of multimorbidity either in Aboriginal or non-Aboriginal patients.

**Table 2:** Patient level characteristics as predictors of multimorbidity, defined as two or more chronic diseases: Results from generalised linear modelling.

	Aboriginal cohort		Non-Aboriginal cohort	
	≥ 2 Morbidities*	≥ 3 Morbidities**	≥ 2 Morbidities*	≥ 3 Morbidities**
	Risk Ratio (95% CI)	Risk Ratio (95% CI)	Risk Ratio (95% CI)	Risk Ratio (95% CI)
**Sex**				
Male	1.0	1.0	1.0	1.0
Female	1.1 (1.0–1.1)	1.2 (1.1–1.2)	1.0 (1.0–1.1)	1.1 (1.1–1.2)
**Age Categories**				
25–39 Years	1.0	1.0	1.0	1.0
40–49 years	1.0 (0.9–1.1)	1.2 (1.0–1.4)	1.3 (1.2–1.5)	1.4 (1.2–1.7)
50–59 years	1.1 (1.1–1.2)	1.3 (1.1–1.5)	1.6 (1.2–2.0)	1.9 (1.6–2.2)
**Area of residence**				
Highly accessible/ metropolitan	1.0	1.0	1.0	1.0
Accessible/Inner regional areas	1.0 (0.9–1.1)	1.0 (0.8–1.1)	1.0 (0.9–1.1)	0.9 (0.8–1.1)
Moderately accessible/Outer regional areas	1.0 (0.9–1.1)	1.0 (0.9–1.2)	1.0 (0.9–1.1)	1.0 (0.9–1.1)
Remote areas	1.0 (0.9–1.1)	1.0 (0.9–1.1)	0.9 (0.8–1.0)	0.8 (0.7–1.0)
Very remote areas	1.1 (1.0–1.2)	1.2 (1.1–1.3)	1.1 (1.0–1.3)	1.0 (0.8–1.3)
**SEIFA quartiles**				
1^st^ quintile (highest disadvantage)	1.0	1.0	1.0	1.0
2^nd^ Quintile	1.0 (0.9–1.1)	1.1 (1.0–1.2)	1.0 (0.9–1.1)	1.0 (0.9–1.1)
3^rd^ Quintile	0.9 (0.9–1.1)	1.0 (0.9–1.1)	1.0 (0.9–1.1)	1.0 (0.9–1.2)
4^th^ Quintile	1.0 (0.9–1.1)	1.1 (0.9–1.2)	0.9 (0.9–1.0)	1.0 (0.9–1.0)
5^th^ Quintile (least disadvantage)	1.0 (0.9–1.1)	1.0 (0.9–1.2)	0.9 (0.9–1.0)	1.0 (0.9–1.1)

* Compared to zero and one condition

** Compared to two or less conditions.

### Patterns of disease clusters

Metabolic disorders like *diabetes* and *hypertension* often clustered in the Aboriginal cohort, mostly associated with *chronic kidney disease*, *alcohol abuse*, *acid peptic diseases*, *heart failure and COPD*. In Aboriginal patients, the expected prevalence of such disease duplets and triplets were more than would be expected by chance from the individual conditions, whereas in non-Aboriginal patients the observed proportions were smaller compared to the Aboriginal cohort (**Table 3**). Additionally, in the non-Aboriginal cohort, *non-skin cancers* and *osteoarthritis* were reported to be present along with other metabolic conditions.

**Table 3:** Most commonly occurring comorbidity patterns in terms of duplets and triplets in ATD cohort.

Duplets				Triplets			
Combination	Observed (O) (%)	Expected (E) (%)	O/E ratio	Combinations	Observed (O) (%)	Expected (E) (%)	O/E ratio
**Aboriginal cohort**							
Diabetes + Hypertension	49.3	28.3	1.7	Diabetes + Hypertension + alcohol abuse	22.9	13.5	1.7
Hypertension + Alcohol Abuse	32.8	28.5	1.1	Diabetes + Hypertension + Chronic Kidney Diseases	22.3	6.6	3.4
Diabetes + Alcohol Abuse	26.6	22.6	1.2*	Diabetes + Hypertension + Acid Peptic diseases	16.6	7.3	2.3
Hypertension + Chronic Kidney Diseases	24.7	13.9	1.8	Hypertension + Acid Peptic Diseases + Alcohol abuse	15.0	7.4	2.0
Diabetes + Chronic Kidney Diseases	24.0	11.0	2.2	Diabetes + Hypertension + Heart Failure	13.9	5.1	2.7
Hypertension+ Acid Peptic Diseases	23.1	15.5	1.5*	Chronic Kidney Diseases + Hypertension + Alcohol Abuse	12.5	6.6	1.9
Diabetes + Acid Peptic Diseases	18.9	12.3	1.5*	Diabetes + Chronic Kidney Diseases + Alcohol Abuse	12.2	5.3	2.3
Acid Peptic Diseases + Alcohol Abuse	18.7	12.4	1.5	Diabetes + Acid Peptic Diseases + Alcohol abuse	12.0	5.8	2.1
Hypertension + Heart Failure	17.2	10.8	1.6	Hypertension + Chronic Kidney Diseases + Heart Failure	11.0	2.5	4.3
Diabetes + Heart Failure	15.2	8.6	1.8*	Diabetes + Hypertension + COPD	10.5	4.4	2.4
**Non-Aboriginal cohort**							
Diabetes + Hypertension	16.0	5.2	3.0	Diabetes + Hypertension + Acid Peptic Disease	4.5	0.8	5.5
Hypertension + Acid Peptic Diseases	11.7	5.7	2.0*	Diabetes + Hypertension + Chronic Kidney Disease	3.7	0.4	10.1
Hypertension + Non-skin cancer	7.2	3.0	2.4	Diabetes + Hypertension + Non-Skin Cancer	2.8	0.4	6.6
Diabetes + Acid Peptic Diseases	5.8	2.2	2.6	Hypertension + Acid Peptic diseases + Non-Skin Cancers	2.6	0.5	5.5
Hypertension + Chronic Kidney Diseases	5.7	2.5	2.2	Diabetes + Hypertension + heart Failure	2.3	0.3	8.3
Non-Skin Cancer + Acid Peptic Diseases	4.9	1.3	3.8	Hypertension + Chronic Kidney Diseases + Acid Peptic Disease	1.9	0.4	4.7
Hypertension + Osteoarthritis	4.5	1.6	2.8*	Hypertension + Chronic Kidney Diseases + Heart Failure	1.7	0.1	13.6
Hypertension + Heart Failure	4.4	1.9	2.3	Diabetes + Hypertension + Osteoarthritis	1.7	0.2	7.4
Hypertension + Alcohol abuse	4.4	3.5	1.2*	Diabetes + Hypertension + Atrial Fibrillation	1.6	0.2	7.2
Hypertension + Atrial Fibrillation	4.2	1.5	2.8*	Diabetes + Hypertension + Alcohol Abuse	1.5	0.5	3.1

*Adjusted odds ratios are not statistically significant from corresponding logistic regression models.

For Aboriginal patients, the LCA identified four classes: *Minimally Diseased* (*MD*) Group (prevalence of all conditions is below cohort average), *Very Sick* (*VS*) Group (above-average prevalence of 17 out of 18 conditions), *Metabolic* Group (excess prevalence of diabetes, hypertension and chronic kidney diseases), and *Drug Abuse-Mental-Respiratory* (*DMR*) Group (excess prevalence of alcohol and drug abuse, depression, COPD, asthma and acid peptic disorder). For the non-Aboriginal cohort, there were six classes including the four described above as well as a *Cancer-Musculoskeletal-Gastric* (*CMG*) Group (excess prevalence of non-skin cancers, osteoarthritis, low back pain and acid peptic diseases) and a *Complex Cardiometabolic* (*CCM*) Group (excess prevalence of atrial fibrillation, heart failure, valvular heart disease, and other associated respiratory and metabolic conditions).

**Fig 2:**
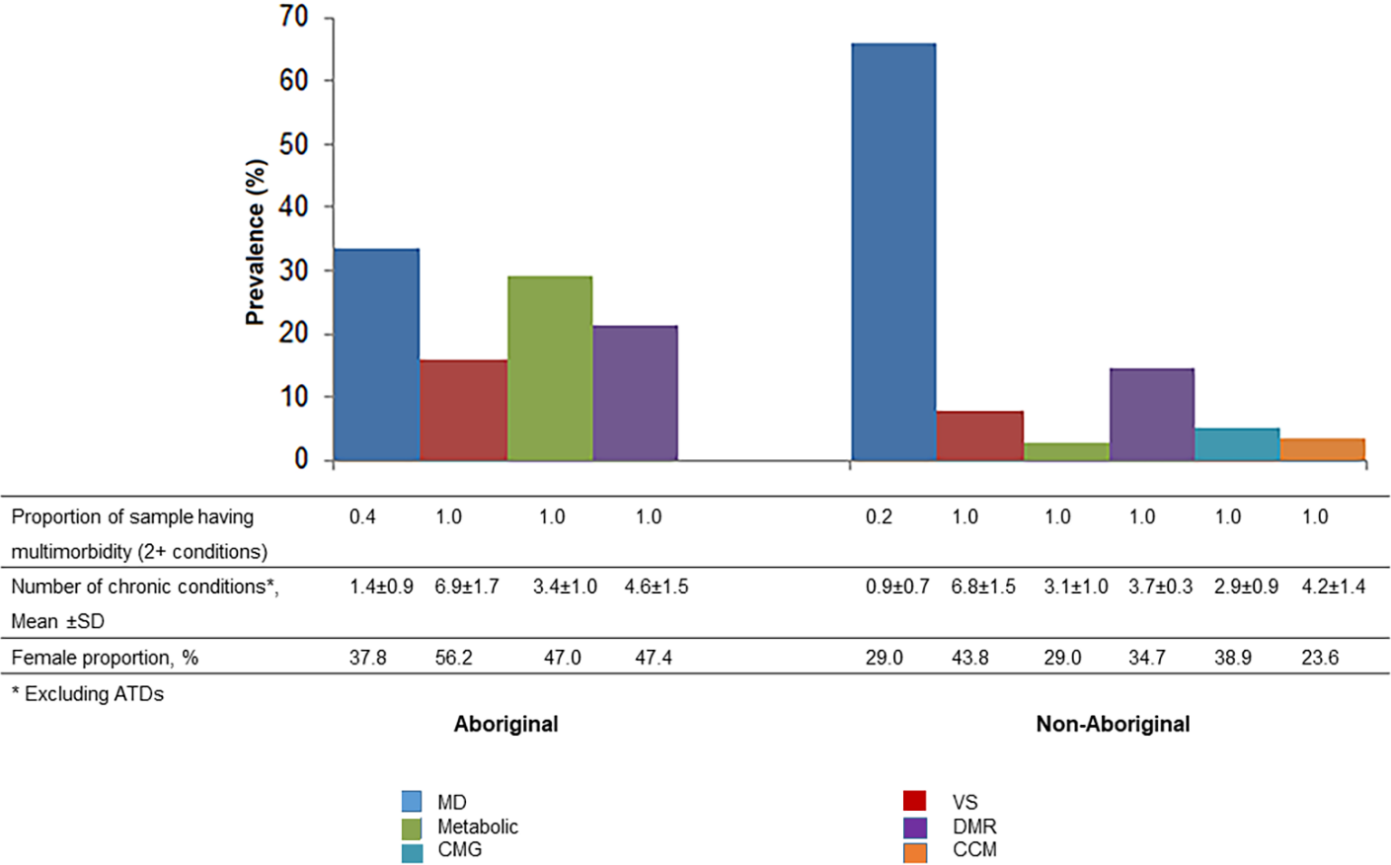
Characteristics of the multimorbidity disease cluster identified through latent class analysis. ATDs: Atherothrombotic diseases; SD: Standard deviation; CMG: Cancer-Musculoskeletal-Gastric; VS: Very Sick; DMR: Drug abuse-Mental-Respiratory; MD-Minimally Diseased; CCM: Complex Cardiometabolic.

The *MD* Group was the largest group (non-Aboriginal cohort: 66% vs. Aboriginal cohort: 33.4%). The majority of member patients in this group had less than two conditions, so only 20–40% of them met our definition of multimorbidity (**Fig 2**). The other groups showed variable numbers of conditions and all group members (100%) had multimorbidity. The mean number of chronic conditions in Aboriginal patients in these other groups ranged from 3.4 in the *Metabolic* Group to 6.9 in the *VS* Group. In contrast, the mean number of chronic conditions in these other groups for non-Aboriginal people varied from 2.9 for the *CMG* Group to 6.8 in the *VS* Group.

For the Aboriginal cohort, the risk of being in non-*MD* multimorbidity groups was significantly higher for women than men (**Table 4**). In particular, Aboriginal women had 90% higher risk of being in the *VS* Group (RRR 1.9; 95%CI: 1.4–2.5) compared to the *MD* Group. The risk of being in the *Metabolic* and *VS* Group were 1.5 times (RRR 1.5; 95%CI: 1.1–2.0) and 1.8 times (RRR 1.8; 95%CI: 1.2–2.6) higher, respectively, for those living in very remote areas compared to those living in the Metropolitan region. Each five-year increase in age increased the risk of being in the *Metabolic* Group and *VS* Group by 30% to 40%, respectively (**Table 4**).

**Table 4:** Factors associated with group member probabilities by latent disease classes—results from multinomial logistic regression (Aboriginal cohort only).

	Minimally Diseased (MD) Group*		Very Sick (VS) Group		Metabolic Group		Drug Abuse-Mental- Respiratory (DMR) Group	
	%	RRR	%	RRR (95% CI)	%	RRR (95% CI)	%	RRR (95% CI)
**Gender**								
Male	62.2	1.0	43.8	1.0	53.0	1.0	52.6	1.0
Female	37.8	1.0	56.2	1.9(1.4–2.5)	47.0	1.4(1.1–1.8)	47.4	1.5(1.1–1.9)
**Age (per 5 year increase)**		1.0		1.4(1.2–1.5)		1.3(1.2–1.5)		1.0(0.9–1.0)
**Area of residence, (%)**								
Metropolitan	35.3	1.0	29.0	1.0	30.1	1.0	34.5	1.0
Inner regional	5.8	1.0	4.2	0.8(0.4–1.7)	5.2	0.9(0.5–1.7)	7.3	1.1(0.8–2.2)
Outer regional	18.9	1.0	14.5	0.9(0.6–1.5)	19.5	1.2(0.9–1.7)	21.7	1.2(0.8–1.7)
Remote/Very remote	40.0	1.0	52.3	1.8(1.2–2.6)	45.1	1.5(1.1–2.0)	36.5	0.9(0.7–1.4)
**SEIFA quintiles, (%)**								
1^st^ quintile	27.8	1.0	27.5	1.0	23.8	1.0	25.3	1.0
2^nd^ Quintile	17.2	1.0	20.6	1.4(0.9–2.2)	21.7	1.6(1.1–2.3)	18.8	1.2(0.8–1.7)
3^rd^ Quintile	19.4	1.0	18.7	1.3(0.8–2.1)	17.8	1.2(0.8–1.7)	20.6	1.1(0.7–1.6)
4^th^ Quintile	24.1	1.0	24.8	1.3(0.8–2.0)	27.1	1.6(1.1–2.2)	22.2	0.9(0.7–1.4)
5^th^ Quintile	11.5	1.0	8.4	1.1(0.5–2.0)	9.5	1.2(0.8–2.0)	13.0	1.2(0.7–1.9)

* Minimally diseased group is the reference.

**Fig 3:**
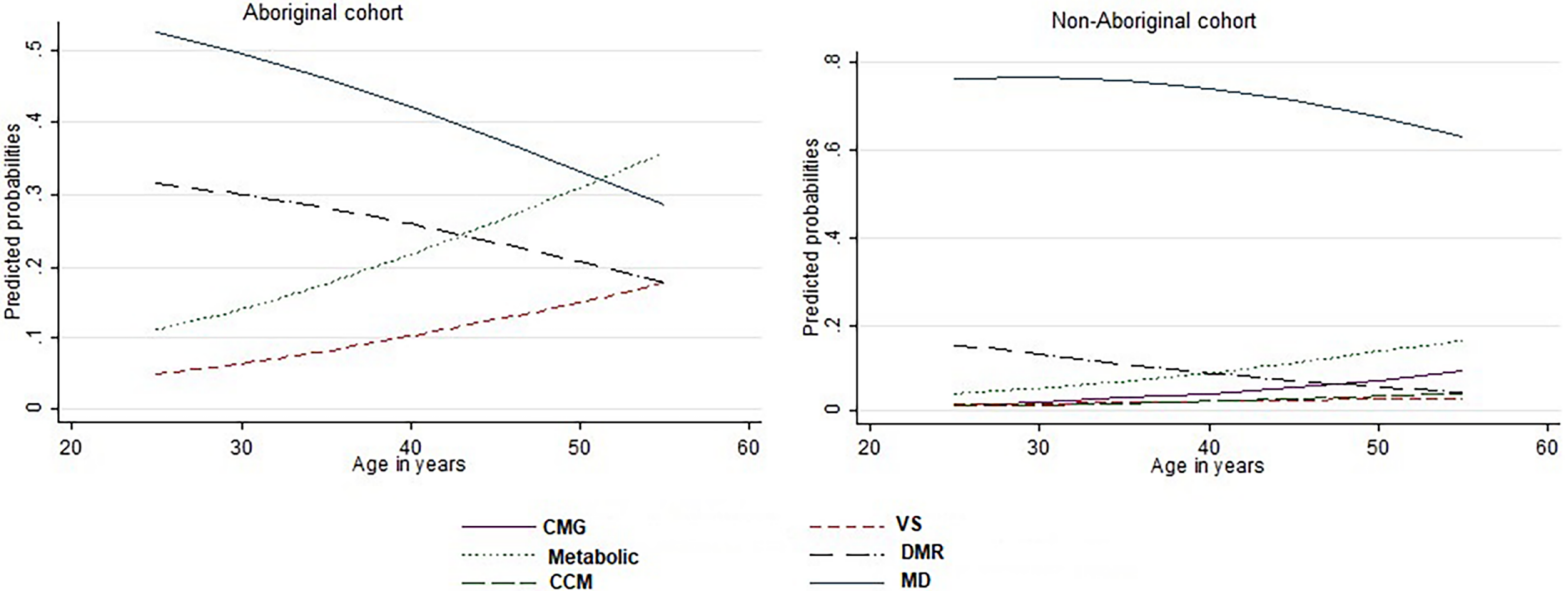
Predicted probabilities of being in different latent classes according to age of the ATD patients. Probabilities are adjusted for gender, SEIFA score and level of remoteness. SD: Standard deviation; CMG: Cancer-Musculoskeletal-Gastric; VS: Very Sick; DMR: Drug abuse-Mental-Respiratory; MD-Minimally Diseased; CCM: Complex Cardiometabolic.

In the non-Aboriginal cohort, the effects of being female and older were similar to that observed in Aboriginal cohort (**Table 5**). However, while being a woman increased the risk of being in *CMG*, *VS* and *DMR* groups compared to the *MD* Group by 20% to 90%, women had a 20% lower risk (RRR 0.8; 95%CI: 0.6–0.9) of being in the *CCM* Group. Age significantly increased the risk of being in the *CMG*, *VS*, *Metabolic*, or *CCM* Groups (**Table 5, Fig 3**). However, with every five-year increase in age, the risk of being in the *DMR* Group reduced by 20% (RRR 0.8; 95% CI: 0.8–0.9). Rurality and remoteness based on ARIA did not have a significant influence on group membership in non-Aboriginal patients.

**Table 5:** Factors associated with group member probabilities by latent disease classes—results from multinomial logistic regression (non-Aboriginal cohort only).

	Minimally Diseased (MD) Group*		Cancer-Musculoskeletal-Gastric (CMG) Group		Very Sick (VS) Group		Metabolic Group		Drug Abuse-Mental- Respiratory (DMR) Group		Complex Cardiometabolic (CCM) Group	
	%	RRR	%	RRR (95% CI)	%	RRR (95% CI)	%	RRR (95% CI)	%	RRR (95% CI)	%	RRR (95% CI)
**Gender**												
Male	70.9	1.0	61.0	1.0	56.1	1.0	70.9	1.0	65.3	1.0	76.4	1.0
Female	29.9	1.0	38.9	1.6(1.4–1.8)	43.8	1.9(1.5–2.3)	29.0	1.0(0.9–1.1)	34.7	1.2(1.1–1.5)	23.6	0.8(0.6–0.9)
**Age (per 5 year increase)**		1.0		1.4(1.3–1.5)		1.2(1.1–1.3)		1.3(1.2–1.4)		0.8(0.8–0.9)		1.3(1.2–1.4)
**Area of residence**												
Metropolitan	77.1	1.0	76.5	1.0	77.6	1.0	77.1	1.0	74.3	1.0	76.1	1.0
Inner regional	8.4	1.0	8.6	1.1(0.8–1.3)	7.7	0.8(0.6–1.2)	7.5	0.8(0.7–1.0)	10.2	1.2(0.9–1.6)	10.4	1.2(0.9–1.6)
Outer regional	9.2	1.0	10.5	1.1(0.9–1.3)	9.5	1.0(0.7–1.5)	9.8	1.1(0.9–1.2)	10.1	1.1(0.8–1.4)	9.3	1.0(0.7–1.4)
Remote/Very remote	5.3	1.0	4.4	0.8(0.6–1.1)	5.2	1.1(0.7–1.7)	5.6	1.0(0.8–1.2)	5.3	1.0(0.7–1.5)	4.2	0.8(0.5–1.3)
**SEIFA quintiles**												
1^st^ quintile	19.1	1.0	20.5	1.0	17.4	1.0	19.4	1.0	19.8	1.0	15.7	1.0
2^nd^ Quintile	11.2	1.0	9.9	0.8(0.6–1.2)	10.2	1.0(0.7–1.5)	11.1	0.9(0.8–1.1)	11.0	0.9(0.7–1.2)	10.7	1.2(0.8–1.7)
3^rd^ Quintile	22.5	1.0	25.9	1.1(0.9–1.2)	17.2	0.8(0.6–1.2)	24.5	1.1(0.9–1.2)	23.1	0.9(0.7–1.2)	26.3	1.4(1.1–1.8)
4^th^ Quintile	26.0	1.0	25.4	0.9(0.7–1.0)	30.8	1.3(0.9–1.2)	25.0	0.9(0.8–1.1)	24.1	0.9(0.7–1.1)	23.8	1.1(0.8–1.5)
5^th^ Quintile	21.1	1.0	18.2	0.8(0.6–0.9)	24.4	1.3(0.9–1.8)	20.0	0.9(0.8–1.1)	21.9	1.0(0.7–1.2)	23.5	1.3(0.9–1.8)

* Minimally diseased group is the reference.

## Discussion

This is the first study to empirically characterise the differences in the broad patterns of multimorbidity in any Aboriginal and non-Aboriginal population groups, and in particular in a relatively younger population. Several methods have been used previously to quantify burden of multimorbidity in different population settings [27, 28, 47], but our study examining multimorbidity in ATD patients has identified and quantified clusters or patterns of diseases between relatively young Aboriginal and non-Aboriginal cohorts aged 25–59 years, and differences between subgroups within each cohort. Overall, we identified that one in two ATD patients admitted to WA hospitals had multimorbidity, with Aboriginal patients having twice the age-adjusted risk of multimorbidity compared to non-Aboriginal patients. Hypertension, diabetes, alcohol abuse disorders, chronic kidney diseases and acid peptic diseases were the leading comorbidities, often linked with the most prevalent major comorbid pairs and complex multimorbid triplets across both Aboriginal and non-Aboriginal cohorts. The LCA identified four meaningful classes of multimorbidity in Aboriginal and non-Aboriginal patients with an additional two distinct clinical clusters in non-Aboriginal people. The largest proportion of patients (33.4% in Aboriginal and 66% in non-Aboriginal patients) were assigned to the “*Minimally Diseased”* Group, with most patients having less than one condition, whereas other groups showed variability in degree and pattern of multimorbidity. The patient groups belonging to different multimorbidity clusters had different disease profiles, and the burden of different chronic morbidities was noticeably different between groups, in terms of the types, numbers and prevalence of diseases. The risk increased with increasing age and remoteness status. Women had a higher risk of complex multimorbidities than men.

The *Metabolic* and *Drug abuse-Mental-Respiratory* groups together included half of the Aboriginal ATD cohort, and were responsible for the maximum multimorbidity burden in the Aboriginal cohort. The complexities of these disease clusters can be inferred from their significantly higher average number of conditions compared with the relatively healthy group (*Minimal Disease Group*). *Metabolic*, *Cancer-Musculoskeletal-Gastric*, *and Drug abuse-Mental-Respiratory* groups comprised more than a quarter of non-Aboriginal ATD cohort. Increasing age was identified as a strong predictor of all complex multimorbidity patterns except for *Drug abuse-Mental-Respiratory* group where the risk of being in this group reduced with increasing age in the non-Aboriginal cohort, but made no difference for Aboriginal patients.

It is important to understand the relevance of a high average number of comorbidities in each individual group. Based on simple disease counts, almost half of the study cohort was identified as having multimorbidity. It has been suggested that the definition of multimorbidity (two or more chronic conditions, as we have done) may over-estimate the proportion of patients with multiple morbidities, and that this may not be useful in differentiating patient needs and help in resource planning [3, 48, 52]. Our approach, using more complex and appropriate methods and based on disease combinations and LCA, identified distinct clusters of diseases in this ATD population. Identification of complex multimorbidity clusters is likely to facilitate recognition of patients with higher care needs, who can be flagged as needing special attention in formulating their care plans and designing secondary and tertiary prevention strategies. Current treatment guidelines are generally based upon trial evidence related to outcomes for one key condition and they fail to take into consideration the impact of associated comorbidities and multiple drug therapies on the treatment outcomes. Knowledge of the specific combinations of chronic conditions highlights the need for reconsideration of clinical guidelines for managing patients with common comorbidity patterns, and may assist policy planners in identifying comprehensive service configurations to address patient needs more appropriately. The differences in multimorbidity patterns across Aboriginal and non-Aboriginal patient cohorts identified in our study also emphasises the need to design differential preventive strategies based on patient characteristics.

It is difficult to directly compare our study findings with those of previous studies conducted in Australia and globally as the results depend on factors like the number and type of chronic conditions considered, the population under study, methods of data collection and types of analyses performed, as well as the demographic and risk factor prevalence in the study population. Despite these caveats, cardiometabolic conditions (like hypertension and diabetes), asthma, arthritis, chronic renal diseases and acid peptic diseases have been found elsewhere to be prevalent conditions which co-occur with other chronic conditions [47, 48, 53, 54]. A systematic review of 14 articles showed considerable methodological heterogeneity in patterns of multimorbidity and identified 63 different patterns composed of three or more diseases [27]. However, those authors found three common patterns across studies, comprising cardiovascular and metabolic diseases, mental health problems, and musculoskeletal disorders [27]. Islam et al. employed multiple analytical approaches to describe patterns or clusters of 10 prevalent conditions in a sample of 4,574 older Australians [47]. They identified four different classes, with the largest group being a minimal disease group (55.5% of cohort), and the other three groups almost matching our *Metabolic*, *CCM*, and *CMG* groups. The similarity of findings with our study, in spite of methodological differences, confirms broad disease clustering in multimorbidity patients over different contexts. A recent analysis using New South Wales linked administrative data has highlighted the disparity in multimorbidity prevalence between Aboriginal and non-Aboriginal hospitalised patients although the patterns of multimorbidity in that patient cohort were not reported [55]

Previous studies exploring the effect of poorer socioeconomic status on multimorbidity demonstrated that increasing deprivation was significantly associated with higher risk of multimorbidity [1, 56]. However, in our study, we failed to demonstrate any independent association between our SES measure (SEIFA) and multimorbidity. The use of the area-based SEIFA score is a poor proxy for SES and could potentially over- or under-estimate the level of disadvantage of an individual [29] especially in Aboriginal patients. The challenges of using SEIFA as a SES measure in health research have previously been highlighted, with researchers cautioned about using it if good measures of SES like education, income and occupation are available at the individual level [43, 57]. This highlights the need for obtaining additional SES measures beside SEIFA in existing linked administrative data so that the influence of socioeconomic status on health outcomes in linked health data can be explored adequately. Nevertheless, the higher prevalence of multimorbidity in our Aboriginal compared to non-Aboriginal cohort can in part be explained by disparities in SES, and the lack of an association between SES (SEIFA) and multimorbidity as reported in our study partly may be because the analyses already stratify on what is likely to be a strong proxy measure for SES.

A major challenge of multimorbidity studies is the use of different diseases which will influence the prevalence and pattern of comorbidities, and how the diseases were ascertained [27, 58]. Our study is based on a finite number of selected chronic conditions and only included information recorded in the HMDC. However, prevalence estimates based on hospitalisation are likely to underestimate the true community burden of multimorbidity in both populations because non-admitted patients were not captured. This burden could potentially affect the extent of disparity shown. Furthermore, there is a possibility of under-coding or over-coding of morbidities in the HMDC given we could not independently validate the selected comorbid conditions. The strengths of this study include the use of linked population-based data; the comprehensive, high-quality data linkage mechanism [59] and use of an evidence-based algorithm for improved identification of Aboriginal patients in the system [42]. This methodology is cost and time efficient in comparison to population surveys and recruitment of participants. Considering the substantially lower life expectancy for Aboriginal Australians compared to non-Aboriginals, selection of a relatively younger age group for this study has ensured adequate representation of Aboriginal patients while also highlighting the burden of multimorbidity in a relatively younger non-Aboriginal population. The 14.5 years of lookback period to identify the comorbidities is likely to have captured virtually all prior disease manifestations requiring hospitalisation [30, 60, 61]. The use of two different analytic methods to identify the patterns of occurrence in a single data set and the high degree of consistency in the results provides confidence in the observed patterns.

Effective and efficient long-term management of individuals with multimorbidity is often challenging [9, 62, 63]. The growing burden of chronic disease has placed more importance on the complex health care needs of these patients who require complex treatment and care, and necessitate special attention, knowledge, and skills of clinicians, nurses and families [63]. Our study demonstrates that these patients consist of groups characterised by distinct disease patterns. Current treatment guidelines overwhelmingly focus on a single disease and the health system is fragmented and sub-specialised [64]. Poor patient-provider communication, limited standard consultation time, lack of care coordination and shared decision-making are important challenges for managing multimorbidity [65, 66]. These challenges are even more marked for Aboriginal people who often fail to receive safe and culturally appropriate health services, and may be exposed to institutional racism, poor communication with health providers and economic hardship [67]. A paradigm shift in how to provide high-quality, patient-centred, holistic care to patients with cardiovascular diseases and multimorbidity is needed, and it needs to include care redesign for patients living in rural and remote areas. Development of care plans based on the individual patient needs, their priorities and lifestyle, and with their unique combination of diseases and environment is essential. Our study highlights the need for differentiated treatment strategies for different disease groups, with efforts to ensure an effective continuum of care from the hospital to the community. The proposed “Health Care Homes”, a federal Government-funded initiative to provide a flexible, comprehensive, coordinated and patient-centred care for patients with complex and chronic conditions [68], offers a potential strategy for better managing these groups of patients but is yet to be successfully implemented to realise its vision. At the population level, prevention and reduction in the burden of multimorbidity may require greater synergy across interventions aimed at reducing individual chronic conditions. However, the dearth of information on how multiple preventive interventions can be delivered holistically and in an effective and efficient manner in an era of increasing subspecialisation makes this challenging. Effective implementation of innovative approaches for early detection of disease and treatment, which includes coordinated care, self-management and chronic disease management plans, is essential for improvements in care management [69] and will be best served by strengthening primary health care. With respect to the higher multimorbidity burden in Aboriginal patients, substantial efforts are needed to design and implement complex chronic disease preventive interventions that are responsive to Aboriginal community needs.

The findings of the present study clearly support the relevance of exploring patterns of multimorbidity, and add to the existing body of evidence on multimorbidity not only in an Australian setting, but also in a global context. More thorough investigation of the potential impact the identified disease clusters have on health care utilization and on patient-related outcomes is needed.

## Supporting information

S1 Table List of possible chronic comorbidities that were identified from hospital admissions data based on ICD-10-AM codes. (PDF)

S2 Table Profile of study participants (prevalent atherothrombotic disease cases): Western Australia 30 June 2014. (PDF)

S1 Fig Overall age-adjusted prevalence of comorbid chronic diseases by sex. (PDF)
