# Supplementary material for: Complexity in disease management: A linked data analysis of multimorbidity in Aboriginal and non-Aboriginal patients hospitalised with atherothrombotic disease in Western Australia
Source: PLoS One. 2018 Aug 14;13(8):e0201496. doi: 10.1371/journal.pone.0201496 (PMC6091927; doi:10.1371/journal.pone.0201496)
Supplement: S1 Table — (PDF) [file pone.0201496.s001.pdf]

S1 Table. List of possible chronic comorbidities that were identified from hospital admissions data based on ICD-10-AM codes.

Disease	ICD-10-AM code
Acid-peptic disease	K21, K2.1, K25 - K29
Alcohol abuse	Z50.2, Z71.4, Z72.1, F10, K70, E24.4, G31.2, G62.1, G72.1, I42.6, K29.2, K86.0, O35.4, T51.9
All Valvular heart diseases (plus complications of valve replacement)	I05-I09, I34-I39, Q22, Q23, T82.0, T82.6, Z95.2, Z95.3, Z95.4
Alzheimer's disease/dementia	F00, F01, F02, F03, G30, G31, F05.1
Anxiety	F40-F41
Asthma	J45, J46
Atrial fibrillation	I48
Chronic kidney disease (CKD)	N00–N08, N11, N12, N14–N16, N18, N19, N25–N28, N39.1, N39.2, Q60–63, T82.4, V56.0, V56.8, V42.0, V45.1, Z49, Z94.0, Z99.2
Chronic liver disease	K70, K71.3-K71.7, K72.1, K73, K74, K75.4
COPD	J40-J44, J47
Crohn's disease	K51
Depression and other mood disorders	F32.0 - F32.2, F32.8, F32.9, F33.0, F33.2, F33.4 - F33.9, F34, F38, F39
Diabetes	E10—E14
Endocrine disorder (except diabetes)	E00-E07, E20-E35
Epilepsy	G40, G41.0, G41.2, G41.9
Heart failure	I50
Hypertension	I10-I15
Low Back pain	M51.1, M54.3, M54.4, M54.5
Lung diseases due to external agents (pneumoconiosis)	J60-J70
Multiple sclerosis	G35
Non-skin cancer	C00-C42, C45-C96, D00-D48
Osteoarthritis	M15-M19
Peripheral neuropathy	G60-G64.
Psychoses (non-organic psychotic conditions)	F20 - F25, F28, F29, F30.2, F31.2, F31.5, F32.3, F33.3
Recreational drug abuse	Z71.5, F12-F16, F18, F19, F11, R78.1-R78.5, T40, Y12, Z50.3, O35.5
Rheumatoid arthritis and other inflammatory polyarthropathies (incl. gout) systemic connective tissue disorders	M05-M14, M71.2, M45, M46.1, M46.8, M46.9, M30-M36
Skin cancer	C43-C44
Tobacco Smoking	Z71.6, Z72.0, F17, T65.2
Ulcerative Colitis	K50
vision loss/visual disturbances	H53-H54
