# Supplementary material for: Complexity in disease management: A linked data analysis of multimorbidity in Aboriginal and non-Aboriginal patients hospitalised with atherothrombotic disease in Western Australia
Source: PLoS One. 2018 Aug 14;13(8):e0201496. doi: 10.1371/journal.pone.0201496 (PMC6091927; doi:10.1371/journal.pone.0201496)
Supplement: S2 Table — (PDF) [file pone.0201496.s002.pdf]

S2 Table. Profile of study participants (prevalent atherothrombotic disease cases): Western Australia 30 June 2014.

		Non-Aboriginal cohort (n=16,088)	Aboriginal cohort (n=2106)	Total (n=18,194)	P value
**Mean age in years (±SD)**		51.5 (±6.5)	48.5 (±7.4)	51.2 (±6.7)	P=0.000
**Median age in years (IQR)**		53 (48-57)	50 (44-55)	53 (48-56)	
**Age group, n(%)**	25-29	151 (0.9%)	35(1.7%)	186 (1.0%)	P<0.0001
	30-34	257 (1.6%)	81 (3.9%)	338 (2.9%)	
	35-39	531 (3.3%)	152 (7.2%)	683 (3.7%)	
	40-44	1333 (8.3%)	295 (14.0%)	1628 (8.9%)	
	45-49	2572 (16.0%)	466 (22.1%)	3038 (16.7%)	
	50-54	4634 (28.8%)	549 (26.1%)	5183 (28.5%)	
	55-59	6610 (41.1%)	528 (25.1%)	7138 (39.2%)	
**Gender, n(%)**	Male	11203 (69.6%)	1148 (54.5%)	12351 (67.9%)	P<0.0001
	Female	4885 (30.4%)	958 (45.5%)	5843 (32.1%)	
**Type of ATDs*, n(%)**	CHD	12448 (77.4%)	1774 (84.2%)	14222 (78.2%)	P<0.0001
	CeVD	3296 (20.5%)	410 (19.5%)	3706 (20.4%)	P<0.0001
	PVD	1251 (7.8%)	158 (7.5%)	1409 (7.7%)	P=0.65
**Metropolitan/rural residence, n (%)**					
Highly accessible/metropolitan		11809 (73.4%)	588 (27.9%)	12397 (68.1%)	P<0.001
Accessible/Inner Regional areas		1425 (8.9%)	163 (7.7%)	1588 (8.7%)	
Mod accessible/Outer regional areas		1579 (9.8%)	388 (18.4%)	1967 (10.8)	
Remote areas		886 (5.5%)	513 (24.4%)	1399 (7.7%)	
Very remote areas		389 (2.4%)	454 (21.6%)	843 (4.6%)	
**SEIFA quintiles, n (%)**					
1st quintile(highest disadvantage)		3090 (19.2%)	530 (25.2%)	3620 (19.9%)	P<0.001
2nd Quintile		1873 (11.6%)	410 (19.5%)	2283 (12.5%)	
3rd Quintile		3682 (22.9%)	405 (19.2%)	4087 (22.5%)	
4th Quintile		4084 (25.4%)	506 (24.0%)	4590(25.2%)	
5th Quintile(least disadvantage)		3359 (20.9%)	255 (12.1%)	3614(19.9%)	
**Hospital type, n (%)**					
Tertiary		4067 (25.3%)	417 (19.8%)	4484(24.6%)	P=0.000
Public Metro		3147 (19.6%)	266 (12.6%)	3413(18.8%)	
Rural public/private		3030 (18.8%)	1332 (63.2%)	4362(24.0%)	
Private Metro		5844 (36.3%)	91 (4.3%)	5935(32.6%)	

*_ not mutually exclusive, IQR=interquartile range, SD-Standard deviation, ATD- Atherothrombotic diseases, CHD-Coronary heart diseases, CeVD- Cerebrovascular diseases, PVD-Peripheral vascular diseases
