# Supplementary material for: Complexity in disease management: A linked data analysis of multimorbidity in Aboriginal and non-Aboriginal patients hospitalised with atherothrombotic disease in Western Australia
Source: PLoS One. 2018 Aug 14;13(8):e0201496. doi: 10.1371/journal.pone.0201496 (PMC6091927; doi:10.1371/journal.pone.0201496)
Supplement: S1 Fig — (PDF) [file pone.0201496.s003.pdf]

S1 Fig. Overall age-adjusted prevalence of comorbid chronic diseases by sex.

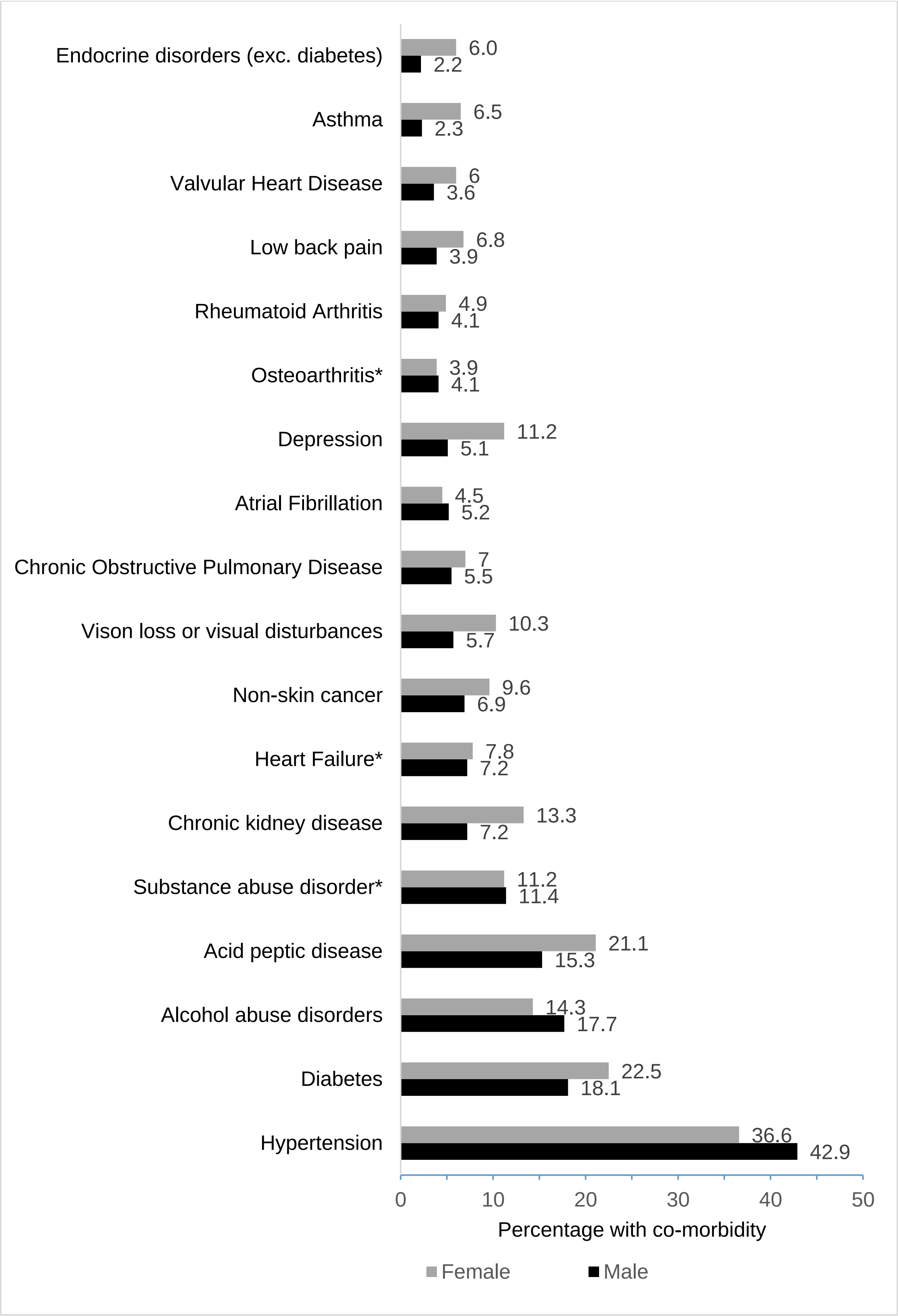

* The differences are not statistically significant
